# Flavonoid Biosynthesis Pathway May Indirectly Affect Outcrossing Rate of Cytoplasmic Male–Sterile Lines of Soybean

**DOI:** 10.3390/plants12193461

**Published:** 2023-10-01

**Authors:** Chunjing Lin, Yuetong Duan, Rong Li, Pengnian Wang, Yanyan Sun, Xiaoyang Ding, Jingyong Zhang, Hao Yan, Wei Zhang, Bao Peng, Limei Zhao, Chunbao Zhang

**Affiliations:** 1 Soybean Research Institute, Jilin Academy of Agricultural Sciences, Changchun 130033, China; lincj@cjaas.com (C.L.); duanyuetong@163.com (Y.D.); rli@cjaas.com (R.L.); pnwang@cjaas.com (P.W.); sunyy@cjaas.com (Y.S.); xyding@cjaas.com (X.D.); zhangjy@cjaas.com (J.Z.); yanhao@cjaas.com (H.Y.); zw0431@cjaas.com (W.Z.); 2 Key Laboratory of Hybrid Soybean Breeding of the Ministry of Agriculture and Rural Affairs, Changchun 130033, China

**Keywords:** soybean, male–sterile lines, RNA–seq, metabolome, outcrossing rates, DEGs, flavonoid pathway

## Abstract

(1) Background: Cytoplasmic male sterility (CMS) is important for exploiting heterosis. Soybean (*Glycine max* L.) has a low outcrossing rate that is detrimental for breeding sterile lines and producing hybrid seeds. Therefore, the molecular mechanism controlling the outcrossing rate should be elucidated to increase the outcrossing rate of soybean CMS lines; (2) Methods: The male–sterile soybean lines JLCMS313A (with a high outcrossing rate; HL) and JLCMS226A (with a low outcrossing rate; LL) were used for a combined analysis of the transcriptome (RNA–seq) and the targeted phenol metabolome; (3) Results: The comparison between HL and LL detected 5946 differentially expressed genes (DEGs) and 81 phenolic metabolites. The analysis of the DEGs and differentially abundant phenolic metabolites identified only one common KEGG pathway related to flavonoid biosynthesis. The qRT–PCR expression for eight DEGs was almost consistent with the transcriptome data. The comparison of the cloned coding sequence (CDS) regions of the *SUS*, *FLS*, *UGT*, and *F3H* genes between HL and LL revealed seven single nucleotide polymorphisms (SNPs) only in the *F3H* CDS. Moreover, five significant differentially abundant phenolic metabolites between HL and LL were associated with flavonoid metabolic pathways. Finally, on the basis of the SNPs in the *F3H* CDS, one derived cleaved amplified polymorphic sequence (dCAPS) marker was developed to distinguish between HL and LL soybean lines; (4) Conclusions: The flavonoid biosynthesis pathway may indirectly affect the outcrossing rate of CMS sterile lines in soybean.

## 1. Introduction

As an annual herb belonging to the family Leguminosae, soybean (*Glycine max* (L.) *Merr*.) is the main source of the seed oil and plant proteins consumed by humans [1]. Compared with corn and rice, research on heterosis started later for soybean, which may partially explain why it has not been exploited for the large–scale production of soybean. In 1985, Davis was the first to report the utilization of cytoplasmic male sterility (CMS) in soybean [2]. After Sun et al. developed the first commercial soybean CMS line in 1993 [3], they subsequently cultivated soybean CMS line YA and its corresponding maintainer line YB 2 years later using a method involving three lines [4]. The first officially approved soybean hybrid, HYBSOY–1, was generated in 2002 and increased soybean production by more than 20% (compared with the control variety) [5].

There has recently been substantial progress in the application of heterosis for soybean production in China, with 42 hybrids developed from 2002 to 2022 [6]. The outcrossing rate, which is the main factor affecting the propagation of soybean male–sterile lines, is closely related to the hybrid seed yield. As a self–pollinated crop, soybean has a low natural outcrossing rate, which leads to unsatisfactory seed yields and seriously restricts the commercial production of hybrid soybean lines [7]. More specifically, hybrid soybean seed yields are lower (unpublished data) than the seed yields of common soybean varieties (2500–3000 kg/hm^2^), making it relatively expensive to produce hybrid soybean seeds on a commercial scale. Therefore, the outcrossing rate of soybean male–sterile lines and the associated molecular mechanism must be thoroughly characterized to breed materials with a high outcrossing rate, decrease seed production costs, and promote the commercialization of hybrid soybean lines.

Soybean is a self–pollinating crop that has typical papilionaceous flowers with complete nectaries [8,9,10]. Weber et al. created 85 soybean hybrids and determined that more than 10% of the F1 plants were higher yielding than their parents, possibly because of the effects of pollinators (e.g., bees or other insects) [11]. Researchers have identified 24 bee species that can pollinate soybean flowers, among which honeybees and leaf–cutting bees are widely used for soybean hybrid seed production [12]. A 3–year pollination experiment [13] confirmed that honeybees, bumblebees, and alfalfa leaf–cutting bees can pollinate soybean flowers.

Flavonoids can regulate floral color, floral fragrance, and fruit flavor in plants [14]. Moreover, flavonoids confer resistance to the biotic and abiotic responses [15]. Regulating the flavonoid synthesis pathway indirectly affects the male fertility by mediating reactive oxygen species clearance under high–temperature stress in soybean [16]. Flavonol, one of the subgroups of flavonoids as UV–absorptive or reflective reagents that could be perceived by insects [17], also contributes to attracting guilds of pollinators such as bees and moths [18,19]. Insect pollinators are attracted to plants with floral colors and scents. Floral colors, which are the most obvious visual stimuli for pollinators, can attract bees and other insects over long distances [20]. The coloration of flowers, fruits, seeds, and other plant tissues and organs is mediated by flavonoids. Because floral colors affect pollinators and insect vectors, the seed coat color influences seed transmission, thereby affecting plant reproduction [21]. In addition, floral scents also serve as bee attractants [22]. In carnations, the inhibited expression of the gene encoding F3H in the anthocyanin biosynthesis pathway increases the release of methyl benzoate [23].

Flower buds with the stigma and upper anthers exposed before blooming can increase the outcrossing rate of rice and cotton, and now the main focus is on the molecular mechanism of the open bud [24,25]. So far, the research on affecting the crossing rate of soybean mainly focuses on the agronomic traits [26]. Few reports exist on a molecular basis for the crossing rate research in soybean. In this study, a male–sterile soybean line with a high outcrossing rate and a male–sterile soybean line with a low outcrossing rate were used for transcriptome sequencing (RNA–seq) and targeted phenol metabolomic analyses, which were conducted to explore the pathways that may affect the outcrossing rate dependent on insect pollinators.

## 2. Results

### 2.1. Transcriptomic Analysis between HL and LL

#### 2.1.1. High–Throughput Sequencing Results

The sequencing of the cDNA libraries constructed using RNA extracted from two soybean male–sterile lines generated 48.18 Gb clean data. Specifically, up to 8.03 Gb clean data were obtained for each sample, with more than 94.12% of the reads having a quality score of Q30. Moreover, 83.76–95.22% of the clean reads for the two male–sterile lines were aligned to the reference genome (Table 1).

#### 2.1.2. Screening for DEGs in the HL vs. LL

**Figure 1:**
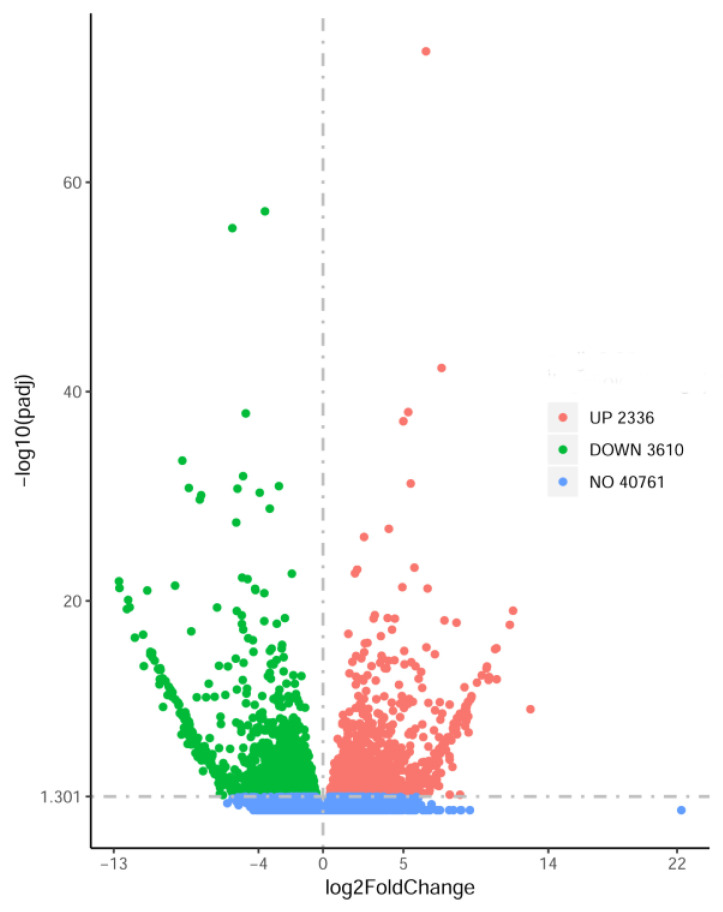
Volcano map of the DEGs between HL and LL. The *X*—axis and Y—axis present the log2(fold—change) values and the adjusted *p*-values, respectively. Pink and green solid circles represent up—regulated and down—regulated DEGs, respectively. Blue solid circles represent genes that were not differentially expressed between HL and LL.

A total of 46,707 expressed genes were detected in the HL vs. LL comparison, of which the expression levels of 2336 and 3610 DEGs were respectively up–regulated and down–regulated in HL (Figure 1).

#### 2.1.3. KEGG and GO Enrichment Analyses

**Figure 2:**
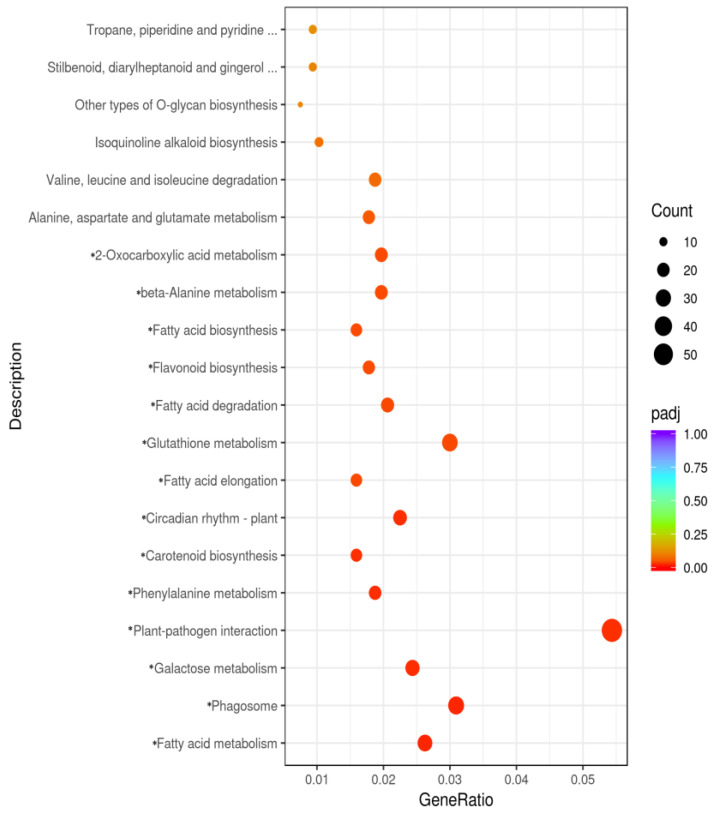
Top 20 KEGG pathways associated with the DEGs between HL and LL. * Significantly enriched KEGG pathway. The *X*—axis and *Y*—axis present the gene ratios and description of the KEGG pathways, respectively. Black circles reflect the number of DEGs. *p*-Values from 0 to 1 are indicated on the color scale from orange to purple, respectively.

Of the KEGG pathways presented in Figure 2, 15 items were significantly enriched among the DEGs detected in the HL vs. LL comparison (Supplementary Table S1).

**Figure 3:**
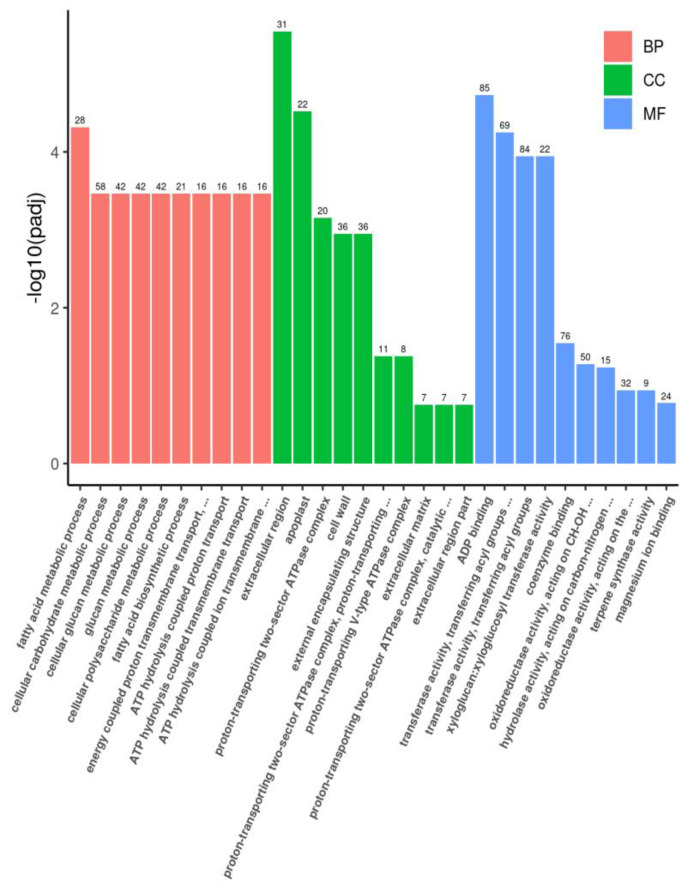
Top 30 significant enriched GO term annotations of the DEGs between HL and LL. The description of GO item (*X*—axis). The value of log10(fold–change) (*Y*—axis); different column shows different category: pink represents biological process (BP), green represents cellular composition (CC), blue represents molecular function (MF), and the number above the column shows the DEGs’ number.

A total of 374 DEGs were associated with the significantly enriched KEGG pathways, including galactose metabolism, flavonoid biosynthesis, and phenylalanine metabolism (Supplementary Table S1). Additionally, 42 enriched GO terms were assigned to 1393 DEGs (Figure 3 and Supplementary Data S1).

### 2.2. Metabolomic Analysis between HL and LL

**Figure 4:**
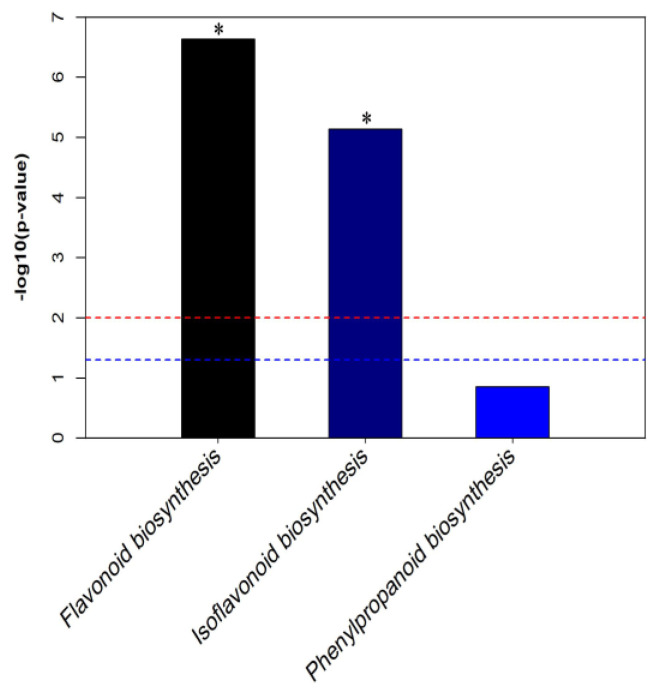
Top 20 KEGG pathways among the differentially abundant phenolic metabolites between HL and LL. * Significantly enriched KEGG pathway. The description of KEGG pathway (*X*−axis), and the value of log10(fold–change) (*Y*−axis); the blue dotted line means *p*-value of 0.05, and the red dotted line means *p*-value of 0.01. * is the significant pathway between HL and LL.

The LC–MS analysis detected 81 of 130 standard phenolic metabolites (Supplementary Data S2 and Supplementary Figure S1). The following 12 metabolites were differentially abundant between HL and LL (Supplementary Table S2): butein, daidzein, ferulic acid, glycitein, isoliquiritigenin, vanillic acid, eriodictyol, genistein, naringenin, procyanidin B2, prunin, and trilobatin. The KEGG analysis indicated that nine differentially abundant metabolites were associated with the following three metabolic pathways: flavonoid biosynthesis, isoflavone biosynthesis, and phenylpropanoid biosynthesis (Figure 4). Five and four of these metabolites were respectively more and less abundant in HL than in LL. Flavonoid biosynthesis and isoflavone biosynthesis were significantly enriched metabolic pathways (Figure 4). Butein, eriodictyol, isoliquiritigenin, naringenin, and prunin were assigned to the flavonoid biosynthesis pathway. Both butein and isoliquiritigenin were more abundant in HL than in LL, whereas eriodictyol, naringenin, and prunin were less abundant in HL than in LL. Daidzein, genistein, and glycitein were assigned to the isoflavone biosynthesis pathway. Daidzein and glycitein were more abundant in HL than in LL, whereas genistein was less abundant in HL than in LL. Vanillic acid, which was assigned to the phenylpropanoid biosynthesis pathway, was more abundant in HL than in LL.

### 2.3. Combined Analysis of the Transcriptomes and Phenol Metabolomes

**Figure 5:**
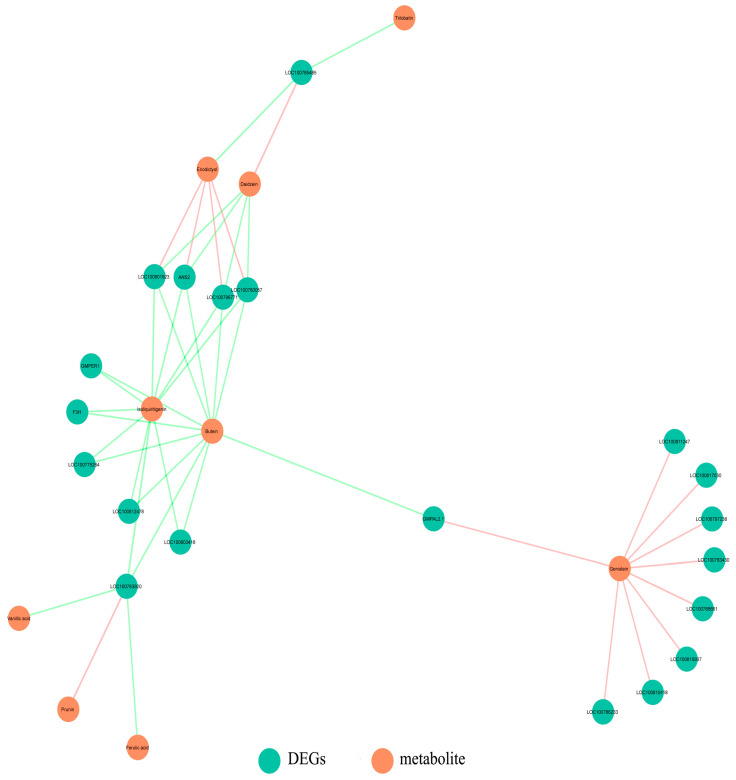
Combined analysis of the significant DEGs in the flavonoid metabolic pathway and the significant differentially abundant phenolic metabolites between HL and LL. Orange and cyan lines indicate positive and negative correlations between the DEGs and metabolites, respectively.

On the basis of the combined analysis of the transcriptome and metabolome data, two metabolic pathways (flavonoid biosynthesis and isoflavone biosynthesis) were screened out in the HL vs. LL comparison, but the flavonoid biosynthesis pathway (gmx00941) was the only common metabolic pathway with significant differences, suggesting it may be the core metabolic pathway affecting the outcrossing rate. The differentially abundant metabolites in the flavonoid biosynthesis pathway included butein, eriodictyol, isoliquiritigenin, naringenin, and prunin (Figure 5).

**Figure 6:**
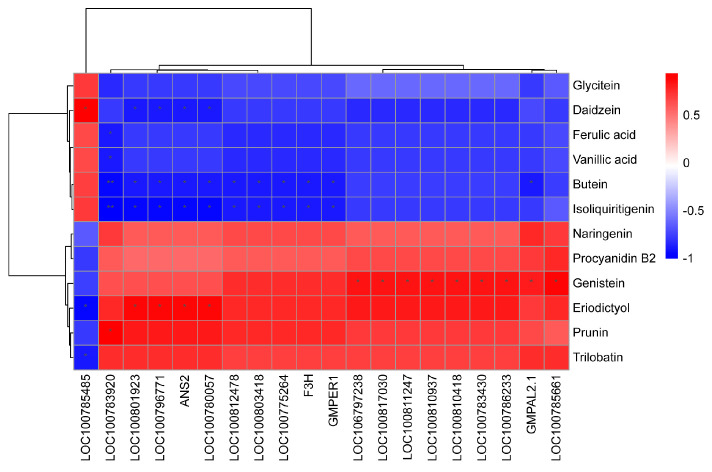
The correlation analysis of DEGs and phenolic metabolite DMs of flavonoid biosynthesis pathway in HL vs. LL. Asterisks significant correlation between HL and LL. The legend from the blue to red means the correlation coefficient from −1 to 1.

The transcriptomic and metabolomic data were also used to evaluate the correlation between the DEGs and the differentially abundant metabolites in the flavonoid biosynthesis pathway (ko00941). In total, 19 of the DEGs were significantly related to the differentially abundant metabolites, of which the expression levels of 3 and 16 genes were respectively higher and lower in HL than in LL. Three DEGs related to isoliquiritigenin and butein had down–regulated expression levels in HL. Similarly, the expression levels of three DEGs correlated with naringenin were lower in HL than in LL. The expression of two DEGs positively correlated with eriodictyol was down–regulated in HL (Figure 5 and Figure 6).

### 2.4. Verification of Key DEGs with qRT–PCR

**Figure 7:**
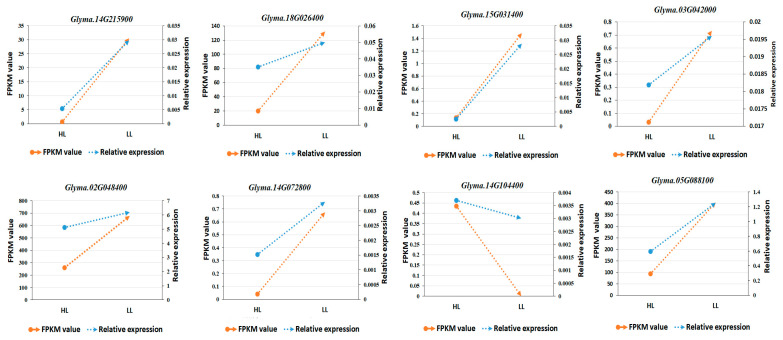
Comparison of the RNA—seq and qRT–PCR data for selected DEGs between HL and LL. Red represents value of FPKM, and blue represents the value of qRT—PCR; round solid point shows the value of HL, and solid triangle shows the value of LL.

Two DEGs involved in the flavonoid biosynthesis pathway (ko00941) were selected for the qRT–PCR analysis. Other DEGs and non–DEGs likely related to other pathways were also included in the qRT–PCR assay. Using Act11 as the reference gene, the expression levels of *Glyma.15G031400* and *Glyma.14G104400* in the phenylpropanoid biosynthesis pathway and *Glyma.02G048400* (*F3H*) in the flavonoid biosynthesis pathway were verified with qRT–PCR. Compared with HL, *Glyma.15G031400* and the *F3H* genes were expressed at higher levels in LL. In contrast, *Glyma.14G104400* was expressed at lower levels in LL than in HL. In addition, the *Glyma.14G215900*, *Glyma.14G072800*, *Glyma.03G042000*, *Glyma.05G088100*, and *Glyma.18G026400* expression trends were almost consistent with the transcriptome sequencing results (Figure 7 and Supplementary Figure S2). The HL vs. LL comparison indicated genes encoding DFR, HCT, ANS, CHS, and FLS were more highly expressed in the male–sterile line with a low outcrossing rate than in the male–sterile line with a high outcrossing rate [27].

### 2.5. Compared CDS Regions between HL and LL

Specific PCR primers were synthesized to amplify the CDS regions of the genes encoding F3H, FLS, UGT, and SUS. The soybean flower bud cDNA served as the template. The PCR products were cloned, after which individual positive colonies were selected for sequencing, which revealed the *FLS*, *UGT*, and *SUS* gene CDS regions were the same in HL and LL. However, seven SNPs were detected in the *F3H* CDS, including six nonsynonymous mutations and one synonymous mutation (Table 2).

### 2.6. Development of Molecular Markers

According to the SNPs within the *F3H* CDS, a dCAPS marker was designed and its utility was assessed with a restriction enzyme digestion (Table 3).

**Figure 8:**
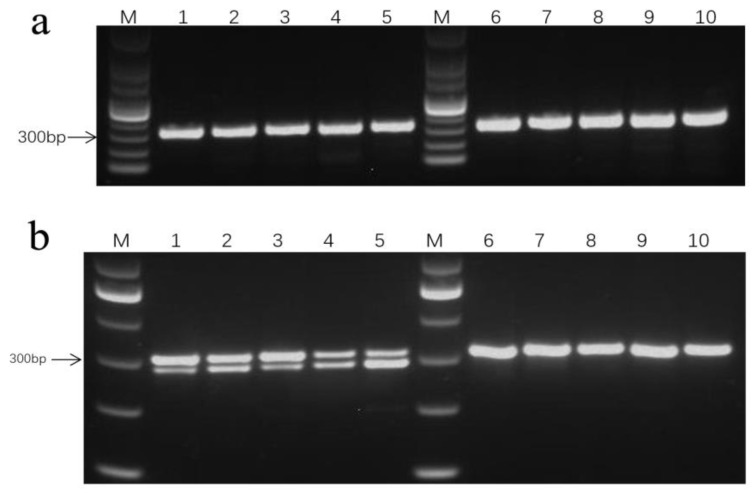
SNP1 marker distinguishing the high–crossing–rate line and the low–crossing–rate line detected by agarose gel. (**a**): Electrophoresis of PCR product of SNP1 site by agarose gel; (**b**): Electrophoresis of enzyme digestion product of SNP1 site by agarose gel. “M”: 100bp marker; 1, 2, 3, 4, and 5 is JLCMS226A, JLCMS89A, JLCMS289A, JLCMS314A, and JLCMS316A, respectively; 6, 7, 8, 9, and 10 is JLCMS313A, JLCMS65A, JLCMS34A, JLCMS295A, and JLCMS84A, respectively.

The marker was named SNP1, and its corresponding partial flanking sequence was tacagcgacaaagtaatggtcagct [heterozygous site with g/g and t] [HL/LL] g [g/c] [HL/LL] aagctcatggaggttgtccgaagcaatggggttagagaaaga. All of the 302 bp fragments amplified with PCR were analyzed using agarose gel electrophoresis (Figure 8a). Because SNP1 was designed according to a specific heterozygous mutation (at 510 bp) (Table 2), the PCR products were digested with *Hind*III, which resulted in three fragments (23, 279, and 302 bp) for LL. The 23–bp fragment was too small to be detected in 1% agarose gels. Thus, in the agarose gels, two fragments (279 and 302 bp) were detected for LL, whereas only one fragment (302 bp) was detected for HL (Figure 8b). The difference in the fragments produced with the enzymatic digestion may be used to distinguish between male–sterile lines with a high outcrossing rate and male–sterile lines with a low outcrossing rate.

## 3. Discussion

Soybean flowers are small and produce heavy and sticky pollen grains, making wind–dependent cross–pollination difficult, even if the flowers have an exposed stigma [28]. Under natural conditions, insect vectors are the main pollinators of various plants. For soybean, bees are the primary pollinators [28,29]. Therefore, the soybean outcrossing rate is affected by whether bees are attracted to the flowers. The floral scent, color, and nectar are bee attractants [30]. In a recent study, caffeine–treated bees made more initial visits to odor–associated target flowers than the other tested bees [31]. Whether other floral compounds affect bee activities and increase the outcrossing rate was undetermined. Hence, we compared a male–sterile soybean line with a high outcrossing rate and a male–sterile soybean line with a low outcrossing rate by conducting RNA–seq and targeted phenol metabolomic analyses to explore the factors potentially influencing the outcrossing rate.

A common pathway related to flavonoid biosynthesis was identified after analyzing the enriched KEGG pathways among the differentially abundant metabolites and DEGs. In this study, the naringenin content was lower in HL than in LL. Of the significant DEGs related to flavonoid biosynthesis, the F3H gene, which was expressed at lower levels in HL than in LL, was related to two differentially abundant metabolites, namely butein and naringenin (Figure 5 and Figure 6). Moreover, among the cloned DEGs related to flavonoids, only the CDS of the F3H gene contained seven SNPs. Earlier research demonstrated that F3H catalyzes the conversion of naringenin to dihydroflavonol, which is the precursor of anthocyanins and flavonols [32]. Therefore, the *F3H* gene encodes a key enzyme in the anthocyanin and flavonoid synthesis pathways [33].

Genes encoding F3H have been cloned in many plants. Recent research on F3H has focused on its regulatory effects on flower and fruit coloration. Other related studies have assessed the effects of abiotic stress on anthocyanin synthesis. In addition, there are also applied studies involving the application of genetic engineering techniques to modify floral fragrances. In carnations, the antisense–based inhibition of F3H gene expression inhibits the anthocyanin synthesis pathway in transgenic plants, resulting in the transfer of metabolites to the benzoic acid pathway, ultimately leading to increased methyl benzoate contents and increasingly fragrant flowers [23]. The soybean floral scent and nectar are important attractants for bees and other insects [34]. Zhang selected 22 “three–line” soybean materials (i.e., male–sterile, maintainer, and restorer lines) and identified the volatile compounds in their flowers via a headspace sampling and gas–chromatography–mass–spectrometry analysis [35]. In total, 31 aromatic volatiles were detected and their relative contents in the flowers of the male–sterile, maintainer, and restorer lines differed significantly. Accordingly, soybean plants may attract pollinators through the regulated production (i.e., contents and proportions) of volatile compounds in flowers, thereby modulating the outcrossing rate. Therefore, the findings of this study imply that decreases in the naringenin content (0.5 times; Supplementary Data S2) and *F3H* gene expression level (0.3–0.8 times; Figure 7) in soybean male–sterile lines with high outcrossing rates will affect the synthesis of certain flavonoids, increase floral metabolite contents through metabolic regulation, and attract bees and other insects, indirectly leading to increases in the outcrossing rate.

## 4. Materials and Methods

### 4.1. Plant Materials

Agronomic traits are correlated with crop outcrossing rates [26]. In this study, soybean RN–type CMS lines [5] JLCMS313A (HL) and JLCMS226A (LL), which have very similar agronomic characteristics (e.g., flower color, plant height, leaf type, branch type, and life cycle; Supplementary Figure S3), were selected as the female plant materials. The male parent used for calculating the outcrossing rate was the strong restorer line JLR259. Ten male–sterile lines with different outcrossing rates were used regarding dCAPS. These lines showed similar agronomic traits with HL and LL. There were five male–sterile lines with a high outcrossing rate (over 60%), JLCMS226A, JLCMS89A, JLCMS289A, JLCMS314A, and JLCMS316A, and five male–sterile lines with a low outcrossing rate (less than 30%), JLCMS313A, JLCMS65A, JLCMS34A, JLCMS295A, and JLCMS84A, respectively. All lines were provided by the Jilin Academy of Agricultural Sciences. In contrast to HL, which is a male–sterile line with a high outcrossing rate (50% in 2021), LL is a male–sterile line with a low outcrossing rate (5% in 2021). Soybean seeds were sown at the Hybrid Soybean Research Base in Fanjiatun. Between 8:00 and 10:00 am on a sunny day, mature flower buds and blooming flowers were collected using tweezers and added to 2 mL centrifuge tubes, which were immediately frozen in liquid nitrogen and stored at –80 °C. The flowers were used for the metabolome analysis, whereas the buds were used for the RNA–seq analysis and for the quantitative real–time polymerase chain reaction (qRT–PCR) analysis of some related genes. Each line was prepared in triplicate.

### 4.2. RNA–seq Analysis

#### 4.2.1. RNA Extraction and Library Construction

Total RNA was extracted from mature flower buds according to the TRIzol precipitation method [36]. The purity of the extracted RNA was checked using the NanoPhotometer^®^ spectrophotometer (Implen, Westlake Village, CA, USA) and by 1% agarose gel electrophoresis. The RNA concentration was determined using the Qubit^®^ RNA Assay Kit and the Qubit^®^ 2.0 Fluorometer (Life Technologies, Carlsbad, CA, USA). The clustering of the index–coded samples was performed using the cBot Cluster Generation System and the TruSeq PE Cluster Kit v3–cBot–HS (Illumina, San Diego, CA, USA) according to the manufacturer’s instructions. The constructed libraries were sequenced using the Illumina HiSeq platform, which generated 150 bp paired–end reads.

#### 4.2.2. Quality Control and Functional Annotation

Clean data were obtained by removing reads containing adapters from the raw data. All of the downstream analyses were performed using the high–quality clean reads. The HTSeq (v0.6.1) program was used to count the reads mapped to each gene in the reference genome (https://www.ncbi.nlm.nih.gov/genome/?term=Glycine+max) (accessed on 22 December 2020). The fragments per kilobase per million (FPKM) value of each gene was calculated according to the gene length and number of reads mapped to the gene and used to estimate gene expression levels. Genes that were differentially expressed between two lines were analyzed using the DEGSeq R package (1.20.0) [37]. The *p*-values were adjusted according to the Benjamini–Hochberg method [38]. A corrected *p*–value of 0.05 and a log2(fold–change) of 1 were set as the thresholds for determining significant differences in expression [39].

#### 4.2.3. Functional Annotation of Differentially Expressed Genes (DEGs)

The Gene Ontology (GO) enrichment analysis of the DEGs was completed using the GOseq R package and the gene length bias was corrected as previously described [40]. The GO terms with a corrected *p*-value less than 0.05 were considered to be significantly enriched. We used the KOBAS software to reveal the significantly enriched Kyoto Encyclopedia of Genes and Genomes (KEGG) pathways (http://www.genome.jp/kegg/)(accessed on 22 December 2020) among the DEGs [41].

### 4.3. Targeted Phenol Metabolomic Analysis

#### 4.3.1. Chemicals

Analytical– or HPLC–grade chemicals and solvents were used. Water, methanol, acetonitrile, and formic acid were purchased from Thermo Fisher Scientific (Waltham, MA, USA), whereas chloroform was obtained from Sinopharm Chemical Reagent Co., Ltd. (Shanghai, China). In total, 130 standards of phenolic metabolites provided by Yuanyebio (Shanghai, China) were used (Supplementary Data S3).

#### 4.3.2. Flavonoid Polyphenol Extraction

Lyophilized flowers (50 mg) were placed in tubes, which contained 600 μL of water/methanol (1/2, vol/vol) as well as 30 ng of succinic acid–2,2,3,3–d4 and Lyso PC17:0 (dissolved in methanol as the internal standard). After adding 400 μL of chloroform, two small steel balls were placed in each tube before the subsequent grinding at 60 Hz for 2 min. The solution was vortexed, ultrasonicated at 4 °C for 20 min, and centrifuged (15,871× *g* at 4 °C for 10 min). The supernatant was collected and a 500 μL aliquot was transferred to a new tube. The above−mentioned steps were repeated using the residue, after which a 300 μL aliquot of the supernatant was added to the first supernatant (i.e., 500 μL aliquot). A 200 μL sample of the combined supernatant was dried in a centrifugal freeze dryer. The lyophilized material was resuspended in a 200 μL mixture comprising methanol and water (7/18, vol/vol) as well as L–2–chlorophenylalanine as the internal standard. The samples were vortexed for 30 s, ultrasonicated at room temperature for 2 min, and centrifuged (15,621× *g* at 4 °C for 5 min). The supernatants (100 μL aliquots) were collected using crystal syringes, filtered through a 0.22 μm microfilter, and transferred to LC vials. The vials were stored at −80 °C prior to the LC–MS analysis.

#### 4.3.3. LC–MS Analysis

The AB ExionLC system (AB SCIEX, Framingham, MA, USA) coupled to the AB SCIEX API 6500 Qtrap+ system (AB SCIEX) was used to analyze targeted phenolic metabolite profiles. Specifically, the positive and negative electrospray ionization modes and the AB SCIEX OS workstation (version 1.7.1) were used. A Waters UPLC HSS T3 column (1.8 μm, 2.1 × 100 mm) was used in the positive and negative ion modes. The binary gradient elution system consisted of (A) water (containing 0.1% formic acid, *v*/*v*) and (B) acetonitrile. The injection volume was 5 μL. Using an electrospray ion source, the analyte was analyzed in the multiple reaction monitoring (MRM) mode for both positive and negative ion scanning, resulting in increased sensitivity. Mass spectrometry parameters, such as the declustering potential and collision energy, were optimized to enable the rapid screening of target compound ion pairs. The optimized mass spectrometry conditions were as follows: positive mode—collision gas: 35, ion spray voltage: 5500 V, ion spray temperature: 600 °C, ion source gas 1: 60, and ion source gas 2: 50; negative mode—collision gas: 35, ion spray voltage: −4500 V, ion spray temperature: 600 °C, ion source gas 1: 60, and ion source gas 2: 50.

#### 4.3.4. Qualitative and Quantitative Analyses

The MRM mode of the triple quadrupole mass spectrometer was used for the quantitative analysis. The mass spectrum data for the two male–sterile lines were obtained. All chromatographic peaks were integrated, and the chromatographic peaks for the same substance in different samples were integrated and corrected. Student’s *t*–test and the fold–change analysis were completed for the two male–sterile lines. The following criteria were used to detect differentially abundant metabolites: *p* < 0.05 and |log2(fold–change)| > 1. The significantly enriched metabolic pathways among the differentially abundant metabolites were determined using the KEGG database.

### 4.4. Combined Analysis of the Soybean Flower Bud Transcriptome and Metabolome

On the basis of the combined analysis of the transcriptome and metabolome data, the differentially abundant metabolites, metabolic pathways, and DEGs affecting the soybean outcrossing rate were identified as follows:

(1) Criteria for screening significant DEGs: |log2(fold–change)| > 1.5 or ˂ 0.67 and *p* ˂ 0.05.
(2) Criteria for screening significant differentially abundant target metabolites: VIP > 1, |log2(fold–change)| > 1.2 or < 0.833, and *p* < 0.05.
(3) The venny2.1.0 online software (https://bioinfogp.cnb.csic.es/tools/venny/) (accessed on 13 August 2021) was used to analyze the common significantly different metabolic pathways identified on the basis of the flower bud transcriptome and floral metabolome.
(4) The KEGG enrichment results for the transcriptome and metabolome were compared to detect common pathways. The gene IDs and metabolites associated with the enriched KEGG pathways were compared with the significant DEGs and differentially abundant metabolites. Finally, the significant DEGs and differentially abundant metabolites were confirmed.

### 4.5. Analysis of DEG Expression with qRT–PCR

Some DEGs detected with the transcriptome analysis were selected for a qRT–PCR assay to verify their expression levels. Total RNA was extracted from soybean flower buds using the Easy Pure Plant RNA Kit (TransGen Biotech, Beijing, China) and then reverse transcribed to cDNA using the EasyQuick RT Master Mix (CWBIO, Beijing, China). Primers specific for the selected DEGs were designed using Premier 3.0 (Supplementary Table S3). The qRT–PCR analysis was performed using the Magics YBR Mixture Kit (CWBIO). The ***Act11*** gene was selected as the internal reference control. Relative gene expression levels were calculated according to the 2^−ΔΔCt^ method [42].

### 4.6. Gene Cloning

The cDNA of male–sterile lines HL and LL was used as the template for the PCR amplification. The Primer–BLAST program was used to design primers specific for the coding sequence (CDS) regions of selected DEGs (Supplementary Table S4). The amplified products were purified using the SanPrep Column DNA Gel Extraction Kit (Sangon Biotech, Shanghai, China) and inserted into the pEASY–Blunt vector (TransGen Biotech) using the pEASY^®^–Blunt Cloning Kit (TransGen Biotech). The inserted fragments were amplified with PCR using the M13F/R primers and then sequenced (Sangon Biotech, Shanghai, China).

### 4.7. Molecular Marker Development

The SNP sites revealed with the sequencing results were used to design derived cleaved amplified polymorphic sequence (dCAPS) markers to distinguish between five RN–type male–sterile lines (JLCMS313A, JLCMS65A, JLCMS34A, JLCMS295A, and JLCMS84A) with a high outcrossing rate (>60% on average) and five RN–type male–sterile lines (JLCMS226A, JLCMS89A, JLCMS289A, JLCMS314A, and JLCMS316A) with a low outcrossing rate (<20%). Above lines are almost the same in the main agronomic traits with HL and LL. All soybean lines were provided by the Soybean Institute of the Jilin Academy of Agricultural Sciences. Genomic DNA was extracted according to the CTAB method [43] and amplified with PCR. The restriction enzyme Hind*III* (Takara, Beijing, China) was used to digest the PCR products.

### 4.8. Statistical Analysis

A test of significance (*p* ≤ 0.05) was performed in R using Student’s *t*–test in the data statistical analysis.

## Figures and Tables

**Table 1:** Summary of the RNA−seq data used for the HL vs. LL comparison.

Samples	Raw Reads	Clean Reads	Clean Base (G)	Q30	GC	Total Map Reads	Total Map Rate
				(%)	(%)		(%)
LL–1	49,522,714	49,284,716	7.39	94.57	45.00	45,185,981	91.68
LL–2	50,578,378	50,327,018	7.55	94.71	44.63	47,697,562	94.78
LL–3	57,316,408	57,061,498	8.56	94.15	44.54	54,336,757	95.22
HL–1	51,070,112	50,849,114	7.63	95.04	45.15	42,589,746	83.76
HL–2	58,457,102	58,255,318	8.74	94.12	44.31	54,765,807	94.01
HL–3	55,662,622	55,402,332	8.31	94.53	44.68	50,317,913	90.82

**Table 2:** SNPs in the CDS region of the F3H gene differentially expressed between HL and LL.

SNP Location	Base to Base	HL	LL	Amino Acid
	(HL to LL)			(HL to LL)
502 bp	G–C	G	C	Gly–Gly
510 bp	G–T	G	G&T	Trp–Leu&Trp
512 bp	G–C	G	C	Glu–Gln
611 bp	C–T	C	T	Pro–Ser
665 bp	G–T	G	T	Gly–Trp
899 bp	G–A	G	A	Asp–Asn
1004 bp	C–T	C	T	Gys–Arg

**Table 3:** Information regarding the dCAPS molecular marker.

Name	Primer (5′–3′)	Enzyme	Product (bp)
	Forward (F)		LL/HL
	Reverse (R)		
SNP1	ACAGCGACAAAGTAATGGGTCAAGC(F)	*HindIII*	302, 279,23/302
	TCTCCAAGATTGACGACGAAGG(R)		

## Data Availability

The data generated in this study were deposited in the NCBI repository, under accession number PRJNA901219 (SRR22298216–SRR22298221).

## Supplementary Materials

The following supporting information can be downloaded at: https://www.mdpi.com/article/10.3390/plants12193461/s1, Figure S1: LC–MS total ion flow overlay chromatogram of HL and LL; Figure S2: Result of relative expression between HL and LL; Figure S3: The agronomic traits of HL and LL. (a) Major traits of HL and LL are shown; (b) the upper row shows three different flowers of HL; the lower row shows three different flowers of LL; Table S1: Significantly enriched KEGG pathways among the DEGs between HL and LL; Table S2: Summary of the significant differentially abundant phenolic metabolites between HL and LL; Table S3: Primers used for the qRT–PCR analysis; Table S4: Primers for gene cloning. Supplementary Data S1; Enriched GO terms assigned to the DEGs between HL and LL; Supplementary Data S2: Values of peak areas for the phenolic metabolites revealed with the LC–MS analysis; Supplementary Data S3: A total of 130 standards in LC–MS.

## Abbreviations

CMS	Cytoplasmic male sterility
qRT−PCR	Quantitative reverse transcription PCR
SNP	Single nucleotide polymorphism
DEG	Different expression gene
FLS	Flavonol synthase
SUS	Sucrose synthase
UGT	UDP−glucosyl transferase
F3H	Flavanone 3−hydroxylase
DFR	Bifunctional dihydroflavonol 4−reductase
HCT	O−hydroxycinnamoyltransferase
ANS	Leucoanthocyanidin dioxygenase
CHS	Chalcone synthase
GO	Gene Ontology
KEGG	Kyoto Encyclopedia of Genes and Genomes
