# Supplementary material for: Flavonoid Biosynthesis Pathway May Indirectly Affect Outcrossing Rate of Cytoplasmic Male–Sterile Lines of Soybean
Source: Plants (Basel). 2023 Oct 1;12(19):3461. doi: 10.3390/plants12193461 (PMC10575370; doi:10.3390/plants12193461)
Supplement: Supplementary file 1 [file plants-12-03461-s001.zip › Supplementary_Material.pdf]

Supplementary Material

Supplementary Figures

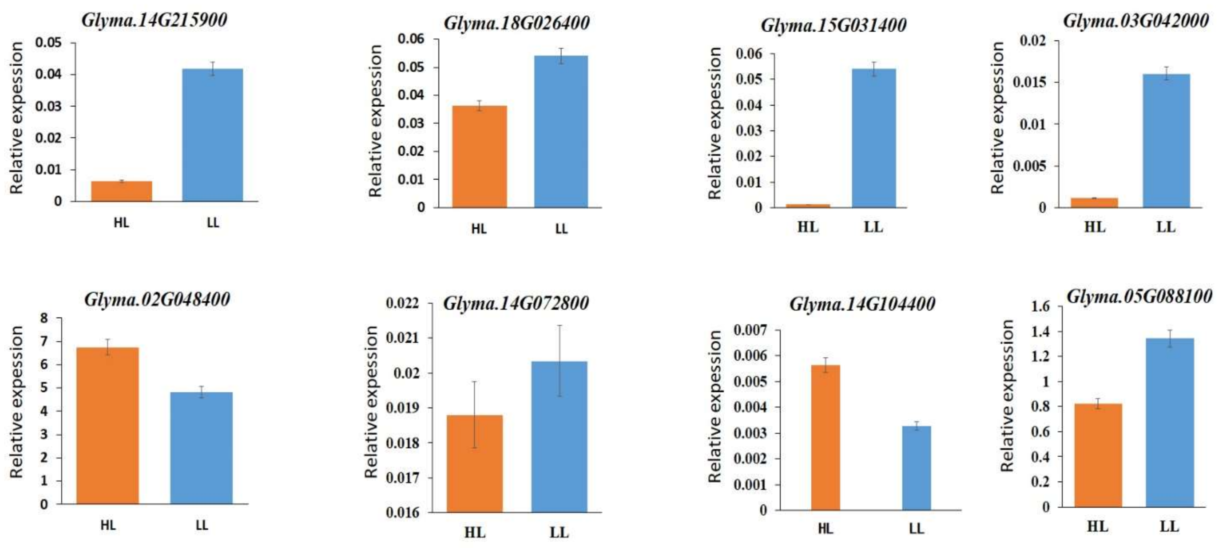

Figure S1. Result of relative expression between HL and LL.

a

Line	Plant height (cm)	Leaf shape	Branch number	Flowering time (M.D)	Mature Time (M.D)	Floral color	Fluff color
HL	107.8	acutifolia	2	6.18	9.15	purple	grey
LL	108.3	acutifolia	1-2	6.18	9.12	purple	grey

b

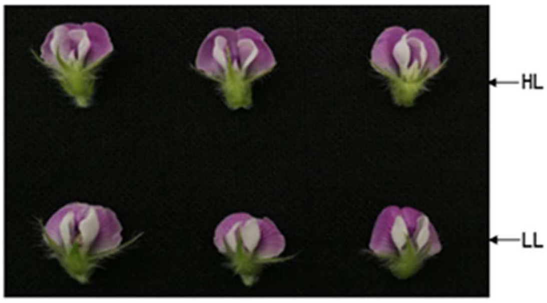

Figure S2. The agronomic traits of HL and LL. (a) major traits of HL and LL are showed; (b) the upper row shows three different flowers of HL; the lower row shows three different flowers of LL.

Supplementary Tables

Table S1. Significantly enriched KEGG pathways among the DEGs between HL and LL

KEGG ID	Description	GeneRatio	P-value	adj.P-value	Count	Up	Down
gmx01212	Fatty acid metabolism	28/1067	0.000109966	0.010385697	28	14	14
gmx04145	Phagosome	33/1067	0.000170257	0.010385697	33	4	29
gmx00052	Galactose metabolism	26/1067	0.000339578	0.013809511	26	7	19
gmx04626	Plant-pathogen interaction	58/1067	0.00050885	0.01438549	58	33	25
gmx00360	Phenylalanine metabolism	20/1067	0.000589569	0.01438549	20	6	14
gmx00906	Carotenoid biosynthesis	17/1067	0.000802181	0.014835704	17	7	10
gmx04712	Circadian rhythm - plant	24/1067	0.000851229	0.014835704	24	12	12
gmx00062	Fatty acid elongation	17/1067	0.002324342	0.034445629	17	2	15
gmx00480	Glutathione metabolism	32/1067	0.002541071	0.034445629	32	22	10
gmx00071	Fatty acid degradation	22/1067	0.002865574	0.034960006	22	13	9
gmx00941	Flavonoid biosynthesis	19/1067	0.003237662	0.035606616	19	3	16
gmx00061	Fatty acid biosynthesis	17/1067	0.004068194	0.035606616	17	8	9
gmx00410	beta-Alanine metabolism	21/1067	0.004086005	0.035606616	21	13	8
gmx01210	2-Oxocarboxylic acid metabolism	21/1067	0.004086005	0.035606616	21	17	4
gmx00250	Alanine, aspartate and glutamate metabolism	19/1067	0.006141126	0.049947826	19	12	7

Table S2. Summary of the significant differentially abundant phenolic metabolites between HL and LL.

Component Name	Category	P-value	adj.P-value	log2(FC)	average(HL)	average(LL)
Butein	Flavanones	0.0111	0.0552	22.68	6.72	0.00
Daidzein	Isoflavones	0.0033	0.0268	1.20	258.88	112.58
Ferulic acid	Phenylpropanoids	0.0003	0.0046	1.20	3940.13	1718.61
Glycitein	Isoflavones	0.0008	0.0077	1.38	198.31	76.30
Isoliquiritigenin	Flavanones	0.0116	0.0552	2.76	97.98	14.47
Vanillic acid	Benzoic acid derivatives	0.0008	0.0077	1.35	4159.62	1635.43
Eriodictyol	Flavanones	0.0004	0.0050	-1.57	37.89	112.80
Genistein	Isoflavones	0.0183	0.0758	-2.41	186.24	987.77
Naringenin	Flavanones	0.0003	0.0046	-1.02	537.87	1087.49
Procyanidin B2	Proanthocyanidins	0.0370	0.1327	-1.22	587.96	1372.84
Prunin	Flavanones	0.0001	0.0046	-1.98	2047.64	8067.45
Trilobatin	Dihydrochalcones	0.0002	0.0046	-21.74	0.00	3.50

Table S3. Primers used for the qRT-PCR analysis

Gene	ID	Forward (5'-3')<br>Reverse (5'-3')	Product length (bp)
Act11	*Glyma.18G290800*	GGTGGTTCTATCTTGGCATC<br>CTTTCGCTTCAATAACCCTA	138
F3H	*Glyma.02G048400*	CTGCCTTCGTCGTCAATCTTGGAG<br>AAGTGGCTATGGACAAACGGCTATG	117
–	*Glyma.15G031400*	TCTGCAATCAAGGATGGTGCAA<br>CCACAAAGTTCATTCCAAAGCGTA	109
–	*Glyma.14G215900*	AGAGGGGGAGTTCTGTGGTAG<br>CAGCCACAAATGCATCACCA	83
–	*Glyma.14G072800*	ATTGTCTCTGTGGAAGGCTGA<br>TTTGTTCTCAGGGTCATTGCAC	124
–	*Glyma.03G042000*	CGTGATTTGCCTTGATTTGTGG<br>GCCCAATCCCATACGAGCAA	115
–	*Glyma.05G088100*	GGAGTTCTTTGCCCTCCCTC<br>AGGAAGAAGGGTGTTTCGGC	194
–	*Glyma.18G026400*	TGAGAAACCCAAGACCCCAA<br>TGCTTGAGACAGAACCTTACAC	139
–	*Glyma.14G104400*	GTGCAAGCCCTTCTGTTCAAG<br>TCCCTTAGCAAGGCAGAATCAG	124

Table S4. Primers for cloning genes

Gene	ID	Forward (5'-3')<br>Reverse (5'-3')	CDS length (bp)	Annealing Temperature (℃)
F3H	*Glyma.02G048400*	CTTACCCATCCTTCAAACTTGAAGCA<br>TGCAGCCAATAATAAAGACAGTGCC	1128	50
FLS	*Glyma.05G088100*	ATGGAAGTGGAAAGAATACAAAC<br>TTATTGGGAAAGCTTATTGAATTT	996	48
UGT	*Glyma.17G019500*	ATGGACTTGAAAGAACAGCCAC<br>TTAAGATTTACGGTCTCTGAATTGT	1410	50
SUS	*Glyma.17G045800*	ATGGCCACTGATCGTTTGACC<br>TTACTCAACAGCAAGGGGCAC	2418	50
